# Supplementary figures and images for: A novel dental biosafety device to control the spread of potentially contaminated dispersion particles from dental ultrasonic tips
Source: PLoS One. 2021 Feb 18;16(2):e0247029. doi: 10.1371/journal.pone.0247029 (PMC7891706; doi:10.1371/journal.pone.0247029)

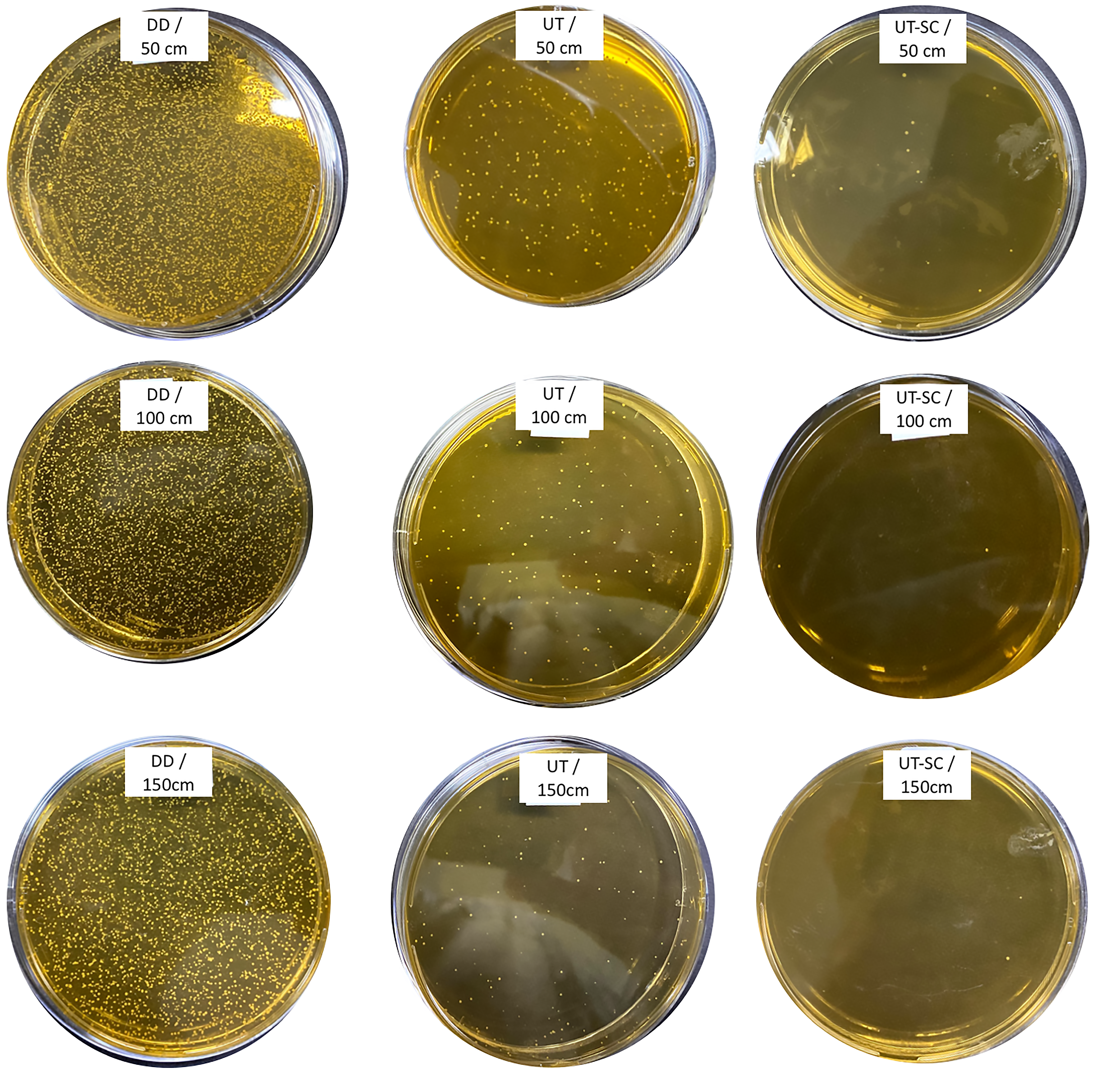

Supplement: S1 Fig — Dental drill (DD) and the ultrasonic tip (UT), as well as the ultrasonic tip combined with the spray control device (UT-SC). (TIF) [file pone.0247029.s001.tif]
